# Factor structure and construct validity of the Adult Social Care Outcomes Toolkit for Carers (ASCOT-Carer)

**DOI:** 10.1007/s11136-015-1011-x

**Published:** 2015-06-03

**Authors:** Stacey E. Rand, Juliette N. Malley, Ann P. Netten, Julien E. Forder

**Affiliations:** Quality and Outcomes of Person-Centred Care Policy Research Unit (QORU), PSSRU Kent, Cornwallis Building, University of Kent, Canterbury, CT2 7NF UK; Quality and Outcomes of Person-Centred Care Policy Research Unit (QORU), PSSRU LSE, Cowdray House, London School of Economics and Political Science, Houghton Street, London, WC2A 2AE UK

**Keywords:** Informal care, Caregiving, Quality of life, Social care, Outcomes, ASCOT, Construct validity

## Abstract

**Background:**

The ASCOT-Carer is a self-report instrument designed to measure social care-related quality of life (SCRQoL). This article presents the psychometric testing and validation of the ASCOT-Carer four response-level interview (INT4) in a sample of unpaid carers of adults who receive publicly funded social care services in England.

**Methods:**

Unpaid carers were identified through a survey of users of publicly funded social care services in England. Three hundred and eighty-seven carers completed a face-to-face or telephone interview. Data on variables hypothesised to be related to SCRQoL (e.g. characteristics of the carer, cared-for person and care situation) and measures of carer experience, strain, health-related quality of life and overall QoL were collected. Relationships between these variables and overall SCRQoL score were evaluated through correlation, ANOVA and regression analysis to test the construct validity of the scale. Internal reliability was assessed using Cronbach’s alpha and feasibility by the number of missing responses.

**Results:**

The construct validity was supported by statistically significant relationships between SCRQoL and scores on instruments of related constructs, as well as with characteristics of the carer and care recipient in univariate and multivariate analyses. A Cronbach’s alpha of 0.87 (seven items) indicates that the internal reliability of the instrument is satisfactory and a low number of missing responses (<1 %) indicates a high level of acceptance.

**Conclusion:**

The results provide evidence to support the construct validity, factor structure, internal reliability and feasibility of the ASCOT-Carer INT4 as an instrument for measuring social care-related quality of life of unpaid carers who care for adults with a variety of long-term conditions, disability or problems related to old age.

## Background

Informal care is an important source of support for people with long-term conditions across the OECD countries [1]. Alongside state- or privately funded social care, unpaid care by friends and relatives meets the needs of adults with illness, disability or frailty associated with old age by providing support with everyday activities and personal hygiene. The balance between formal and informal care varies by country and is influenced, at least in part, by differences in social care systems [2, 3]. An important policy concern, especially as the projected availability of informal care is expected to decline while demand for social care increases [4, 5], is how to support unpaid carers in their caring role. This is particularly relevant given the evidence that high-intensity caregiving may adversely affect carers’ health and well-being [6–9] even if carers may also report positive aspects of caring for a friend or relative [10].

In this context, policymakers in many European countries are at various stages of engaging with the question of how best to support carers [11, 12]. There are some countries where policy strategy for the support of carers is already relatively well developed: for example, in England, informal carers have been recognised as vital to support the quality of life (QoL) of adults with long-term conditions [13, 14]. Policymakers have identified the priority areas of carers’ health and well-being and their ability to sustain a life alongside caring and to participate in education or employment [13, 15]. Indeed, the Care Act (2014) aims to improve carers’ access to support services, such as support to remain in employment, support groups or information and advice.

With limited resources to support long-term care systems, however, especially in the context of a projected increase in demand for long-term care in Europe [3], an important concern is how to effectively allocate resources within long-term care systems to support both adults with long-term conditions and their unpaid carers. While such decisions are often made with limited evidence, there has been increasing interest in the measurement of the quality of life outcomes of social care; such measures may enable policymakers, providers and practitioners to evaluate the effectiveness, quality and value of policy or specific interventions and to determine the most appropriate allocation of resources [16]. Although there are a range of instruments that measure carers’ health, experience, well-being, stress or burden [17–19], the effect of social care support on quality of life (QoL) may not be appropriately captured by such instruments. Inappropriate measures could lead to the effects of policy or interventions being missed. This highlights the need for an instrument designed to measure the effect of social care support or the ‘social care-related quality of life’ (SCRQoL) of informal carers [16, 20].

The purpose of this paper is to assess the feasibility, factor structure, internal consistency and construct validity of a new measure of carer QoL with specific relevance to social care, the ASCOT-Carer. The ASCOT-Carer measures social care-related quality of life (SCRQoL) across seven domains (see Table 1) and has been developed alongside the preference-weighted ASCOT-INT4 instrument to measure SCRQoL of users of social care services [21–23]. This article builds on the content validation and preliminary psychometric analyses conducted during the development of the carer social care-related quality of life measure [20, 24, 25] to establish the psychometric properties of the four response-level measure.

**Table 1:** Carer social care-related quality of life domains

Domain	Definition
Occupation	Being sufficiently occupied in a range of meaningful, enjoyable activities whether it be formal employment, unpaid work, caring for others or leisure activities
Control over daily life	Choosing what to do and when to do it, and having control over their daily life and activities
Self-care	Feeling able to look after oneself, in terms of eating well and getting enough sleep
Personal safety	Feeling safe and secure, where concerns about safety can include fear of abuse or other physical harm or accidents, which may arise as a result of caring
Social participation	Being content with their social situation, where social situation includes the sustenance of meaningful relationships with friends and family, as well as feeling involved and part of their community
Space and time to be yourself	Having space and time in everyday life. Enough time away from caring to have a life of their own outside of the caring role
Feeling supported and encouraged	Feeling encouraged and supported by professionals, care workers and others, in their role as a carer

## Methods

### Development of the ASCOT-Carer

A study conducted in 2007–2008, which drew on two focus groups with care managers and four focus groups with 21 informal carers recruited via carers’ support groups and organisations in Kent, identified seven domains of social care-related quality of life from the carer’s perspective (see Table 1) [25, 26]. The researchers, with support from an advisory group of informal carers and employees of a local authority with adult social care responsibilities, developed a draft questionnaire. The questions were tested in 56 interviews with informal carers using cognitive interviewing methods [27]. This study produced a three response-level version, which is included in the national Personal Social Services Survey of Adult Carers in England (PSS SACE) [28].

The data collected from the 2009/2010 national survey of informal carers known to local authorities in England were analysed to identify the items to include in the final QoL instrument and to establish their psychometric properties [20]. Subsequent cognitive interviewing with 31 carers, which was conducted in 2012 across three local authorities in England, informed minor amendments to the question wording and domain definitions, as well as establishing a four response-level version of the instrument (ASCOT-Carer INT4). This development work is reported in detail elsewhere [29]. The full instrument can be downloaded from the ASCOT website (www.pssru.ac.uk/ascot).

### Analysis Sample and Data Collection

A sample of carers was recruited in 22 of the 150 local authorities in England with adult social care responsibilities who were participating in the Identifying the Impact of Adult Social Care (IIASC) study. These local authorities included representatives of all English Government Office regions (with the exception of the East Midlands) and a range of types: shire counties (11); metropolitan districts (6); London Boroughs (3) and unitary authorities (2).

The carers were recruited via adults with physical disabilities or sensory impairment, mental health conditions or intellectual disabilities who were in receipt of fully or partly publicly funded community-based social care support (e.g. home care, day centre, equipment, meals service) and consented to and participated in an interview for the IIASC study. The users of social care services were asked whether anyone helped them with activities of daily living using questions from the Health Survey for England [30]. If the respondent identified that they received help from friends, family or neighbours, then the respondent was asked at the end of the interview to pass on a letter of invitation to their primary informal carer. (Primary informal carer was defined as a friend, neighbour or relative who spent the most number of hours per week helping the person who had participated in the IIASC interview with activities of daily living (ADL) or instrumental ADL (IADL)). The 990 interviews with social care recipients identified 739 informal carers. The respondent agreed to pass an invitation letter to their carer in 510 cases (69.3 %). Of those carers who received an invitation letter, a total of 387 (75.7 %) interviews with eligible carers were completed.

The interviews with carers were conducted between June 2013 and March 2014 using computer-aided personal interviews conducted either face-to-face in people’s homes or by telephone. Written or verbal informed consent was obtained before the interview. Ethical approval was obtained from the social care research ethics committee.

### Questionnaire

Social care-related quality of life was measured using the ASCOT-Carer four response-level interview (INT4) [29]. The response options in the ASCOT-Carer INT4 correspond to the carers’ ‘ideal state’ (3), ‘no needs’ (2), ‘low level needs’ (1) and ‘high-level needs’ (0) within each of the seven SCRQoL domains to form an overall score between 0 and 21. Carers were asked to rate each domain of quality of life with respect to their current situation.

Various measures of carer experience, health-related quality of life (HRQoL) and quality of life were also included in the interview. The Carer Strain Index (CSI) [31] is a 13-item measure of the strain related to caregiving. The carer is asked to indicate whether (1) or not (0) they have had difficulties with different aspects of caregiving. The Carer Experience Scale (CES) is a preference-weighted measure of caring experience for use in economic evaluations of health and social care interventions [32–34]. The instrument comprises six attributes associated with the experience of caring with three levels of response per attribute. HRQoL was assessed using the EuroQOL-5D (EQ-5D 3L) scored with UK preference values (UK TTO) [35–37]. Overall quality of life was evaluated using a seven-point Likert scale.

The following data were collected from the carer interview: age; sex; employment status; self-reported health; co-residence with the care recipient; and satisfaction with social care support. The suitability of the home for caregiving was assessed using a self-report question with four levels of response developed in earlier work [29]. The UCLA three-item loneliness scale [38] was included as a measure of social isolation. Three items from the Minimum Data Set Cognitive Performance Scale [39] to rate the care recipient’s short-term memory, cognitive skills for daily living and communication, as well as two additional items to capture the care recipient’s disorientation and frequency of behaviours that the carer finds challenging, were also collected. Different aspects of the caregiving situation, such as the duration of caregiving, motivation for providing informal care, number of hours per week of care, the types of care tasks undertaken and the effect of caring on health and employment, were captured using items from the Household Survey of Carers in England 2009/2010 [40]. Socio-demographic data (i.e. age, sex) collected in the care recipient interviews were also linked to the responses to the carer interviews.

### Analysis

Analyses were conducted in Stata version 12. The purpose of the analysis is to evaluate the feasibility, factor structure, internal consistency and construct validity of the ASCOT-Carer. Feasibility, or the acceptability of the items to carers, will be evaluated in this study by reviewing the proportion of missing values per item. The internal consistency across the seven items in the ASCOT-Carer is assessed using Cronbach’s alpha [41], which we interpret as an indicator of reliability.

#### Factor structure

Exploratory factor analysis (EFA) conducted in a previous study on a sample of 35,615 informal carers surveyed across 90 councils in England proposed a one-factor (single scale) solution for the seven-item, three-level version of the carer SCRQoL measure [20].^1^ To evaluate whether the seven domains of the four-level instrument measures a single common underlying construct, a one-factor model was applied using confirmatory factor analysis (CFA) with maximum likelihood estimation. Model fit was assessed using the standardised root mean square residual (SRMR), the root mean square error of approximation (RMSEA), the incremental fit statistics (Tucker–Lewis Index (TLI) and Comparative Fit Index (CFI)) and the parameter estimates. The model fit cut-off values for acceptability were taken to be a RMSEA of ≤ 0.06 (upper confidence interval of ≤ 0.08), SRMR ≤ 0.08 and CFI/TLI ≥ 0.95 [42].

#### Construct validity

The construct validity of the instrument to measure carer ‘social care-related quality of life’ is evaluated using regression analysis. Construct validity is based on testing hypotheses of how a measure should behave in relation to other measures or other factors hypothesised to be associated with the measurement construct [43]. The construct validity of the ASCOT-Carer instrument was assessed using convergent validation, which evaluates the extent to which the construct of the ASCOT-Carer measure correlates with different instruments that measure the same or similar constructs [44]. As there are no other instruments that measure carers’ SCRQoL, the convergent validity of the ASCOT-carer was studied by the association between SCRQoL score and instruments that measure the following associated constructs: the subjective effect of caregiving (CSI [31]), health-related quality of life (EQ-5D [35, 36]), the carers’ experience of caregiving (CES) [32–34]) and overall quality of life (single seven-point item). Pearson’s correlation coefficients were used to study the bivariate associations between the ASCOT-Carer score and these instruments.

Another aspect of construct validity describes the extent to which a measure relates to other variables, such as background characteristics (e.g. age, gender) [45]. This was investigated by studying the relationship between overall ASCOT-Carer SCRQoL scores and characteristics of the carer, the nature of the caring relationship and the care recipient. The hypothesised relationships between SCRQoL and other variables are outlined in Table 2. Associations were initially explored using one-way analysis of variance (ANOVA). Multivariate associations were then analysed with ordinary least squares (OLSs) of the ASCOT-Carer score and the characteristics of the carer, the care recipient and the care situation. Respondents with missing values for the dependent or any of the independent variables were excluded (*n* = 20).

**Table 2:** Expected associations with characteristics of the carer, the care recipient and the caregiving situation

Variable	Expected associations
Carer’s gender	A positive association between male carers and higher quality of life was anticipated. There is evidence for lower quality of life and health outcomes for female compared with male carer [8, 48–50], although this may be mediated by the amount and type of informal care [8]
Carer’s age	An association between older carers and better SCRQoL was expected based on evidence that supports such an association [51–53], particularly in relation to social participation [54]
Carer in paid employment	Carers in employment were expected to be positively associated with the attributes of *Social participation, Control* and *Occupation*, as employment may provide opportunities for meeting others, having more independence and meaningful activity. Carers who are in retirement [55] or are not in work [48] have been found to report better health-related quality of life, so a negative association was expected
Carer self-rated health as bad or very bad	Due to the close relationship between health and general quality of life, a negative association was expected between poor self-related health and ASCOT SCRQoL score
Carer’s UCLA three-item loneliness scale [38]	Loneliness has been found to be associated with a lack of social contact or support and overall QoL, particularly among older caregivers [56]. Therefore, a negative relationship between rating of loneliness and all ASCOT-Carer domains was expected
Care recipient self-rated health as bad or very bad	The care recipient’s health is an indicator of their social care need. Worse physical or psychological health has been found to be associated with increased carer burden or strain and lower QoL [8, 48, 49, 57]. Therefore, a negative association between care recipient poor health and SCRQoL was expected
Carer/care recipient co-residence	Informal carers who live in the same house as the care recipient, especially spouses, reported higher involvement in caregiving tasks and more ‘role captivity’ than carers who live apart from the care recipient [58]. Therefore, co-residence was expected to be associated with lower SCRQoL
Minimum data set cognitive performance scale items [39]; challenging behaviour	Based on evidence that problematic behaviour [58] and impaired cognitive ability [8, 48, 49] are associated with increased carer burden or strain and worse psychological health or well-being, it was anticipated that there would be a negative association with SCRQoL for the items that capture, short-term memory impairment, communication difficulties, disorientation, impaired cognitive skills for daily living and behaviour that the carer finds challenging
Duration of caregiving	Previous studies have found carers’ QoL to be negatively associated with the duration of caring [57, 59]. A negative association between the duration of caregiving and SCRQoL was therefore expected
Hours of care per week	The quality of life of carers was found to be inversely associated with the amount and daily frequency of caring [57, 59]. A negative association between the hours of care per week and QoL was therefore expected
Care tasks—personal care and giving medicines	Personal tasks, such as washing, or those associated with increased anxiety, such as administering medicines or medical procedures, are reported as more burdensome than non-personal tasks, such as transportation or housework [60]. Therefore, help with these two tasks were expected to be associated with lower SCRQoL
Rating of suitability of home design for caring	A worse rating of the design of the home was expected to be associated with lower quality of life, since inadequate home design may increase the reliance of the care recipient on the informal carers’ help and also increase the risk of accidents or physical harm associated with caregiving
Caring has had no effect on health	The aim of social care is to support the health and well-being of care recipients and their carers. Therefore, a positive association was expected between items that capture no impact of caregiving on health and ASCOT-Carer SCRQoL score
Motivation for caring: no one else available; or, the care recipient would not want anyone else to help	The motivation or reason for caring has been associated with quality of life and health outcomes for carers and care recipients [61–64]. Specifically, high extrinsic (i.e. social obligation or expectations) and low intrinsic (i.e. related to personal belief or values) motivations for caring are associated with higher carer burden and anxiety/depression [62, 64]. A negative association between these two extrinsic motivations and SCRQoL was therefore expected
Effect of caring on social/leisure activities, employment or financial situation	The impact of caregiving on everyday life, such as the impact on employment, household income and financial difficulties, may contribute to the stress or burden felt by carers [65, 66]. The aim of social care is to support informal carers to continue a life alongside caring by supporting carers to continue in employment and with social/leisure activities [13, 14] and to avoid significant financial difficulties due to caregiving: therefore, a negative association was expected between items that capture a negative impact of caregiving on time for social/leisure activities, employment or financial difficulties and overall ASCOT-Carer score
Carer rating of satisfaction with services	A negative association was expected between not being satisfied with social care services (i.e. neither satisfied nor dissatisfied, or dissatisfied) and overall ASCOT-Carer score
Survey administration	The administration of surveys by telephone compared with face-to-face may result in systematic differences in response due to differences in social desirability bias by survey administration type, or other factors [67]. A meta-analysis found only small differences between telephone and face-to-face interview responses [68]. In one study, respondents aged over 60 years tend to rate higher levels of anxiety and depression on the GHQ-12 by telephone compared with face-to-face interviews [69]. We therefore expect the difference between telephone and face-to-face interviews to be small with a weak negative association between completion of the interview by telephone and overall ASCOT-Carer score

## Results

The characteristics of the study sample are summarised in Table 3. There was a majority of females (58.9 %), which is slightly lower than the estimate that 60 % of informal carers in England are women [40]. Only 26.4 % of the samples were in paid employment, which is lower than the estimated national figure (46 %) [40]. The sample profile of employment may be partly linked to the high proportion of carers retired from paid employment (46.2 %) compared with only 27 % of carers in England [40]. In the sample, 60.1 % of carers provided 35 or more hours of care per week. This is comparable to those carers known to local authorities in England, but is higher than the national estimate (30 %) [40]. Although there are differences between the study sample and the population estimate of carers in England, the study sample is comparable to the profile of carers in England known to local authorities [40], which represent the carers who are most likely to access social care services or be in need of support or interventions.

**Table 3:** ASCOT-Carer INT4 SCRQoL score by characteristics of informal carers, care recipients and caregiving situation (*n* = 387)

	Frequency	% of total (*n* = 387)	ASCOT-Carer SCRQoL Mean	ANOVA F Statistic^a^
*Carer’s sex (n* = *387)*				
Female	228	58.9	12.9	6.49*
Male	159	41.1	14.2	
*Carer’s age (n* = *387)*				
18–64 years	221	57.1	13.6	0.63
≥65 years	166	42.9	13.2	
*Carer in paid employment* *(n* = *387)*				
No	285	73.6	12.9	13.60***
Yes (FT or PT)	102	26.4	14.9	
*Carer’s self*-*rated health (n* = *387)*				
Very good, good or fair	323	83.5	14.1	50.52***
Bad or very bad	64	16.5	9.81	
*UCLA loneliness: Carer lacks companionship? (n* = *387)*				
Hardly ever or never	234	60.5	15.1	53.67***
Some of the time	101	26.1	11.7	
Often	52	13.4	9.3	
*UCLA loneliness: Carer feels left out? (n* = *387)*				
Hardly ever or never	239	61.7	15.3	75.89***
Some of the time	102	26.4	11.1	
Often	46	11.9	8.7	
*UCLA loneliness: Carer feels isolated? (n* = *387)*				
Hardly ever or never	223	57.6	15.4	68.94***
Some of the time	109	28.2	11.6	
Often	55	14.2	9.1	
*Care recipient sex (n* = *383)*				
Female	212	55.4	13.9	n/a
Male	171	44.6	12.8	
*Care recipient’s age (n* = *383)*				
<65 years	198	51.7	13.1	n/a
≥65 years	185	48.3	13.8	
*Care recipient’s self*-*rated health (n* = *383)*				
Very good, good or fair	277	72.3	14.1	23.34***
Bad or very bad	106	27.7	11.6	
*Live with care recipient?* *(n* = *387)*				
No	90	23.3	16.0	38.04***
Yes	297	76.7	12.6	
*Does care recipient have a short*-*term memory problem? (n* = *387)*				
No	221	57.1	14.3	19.15***
Yes	166	42.9	12.3	
*Is the care recipient disorientated? (n* = *385)*				
No	205	53.0	14.8	43.18***
Yes	180	46.5	11.8	
*Care recipient’s cognitive skills for daily living (n* = *387)*				
Independent, some or moderate difficulties	319	82.4	14.0	26.17***
Severely impaired	68	17.6	10.9	
*Care recipient communication difficulties (n* = *387)*				
No, is understood	176	45.5	14.6	20.70***
Yes, is usually, rarely or never understood	211	54.5	12.5	
*Does care recipient have behaviours that the carer finds challenging? (n* = *387)*				
Never, unusually or sometimes	351	90.7	13.9	43.64***
Frequently	36	9.3	8.7	
*Duration of care giving* *(n* = *387)*				
Up to 10 years	184	47.6	13.9	3.19
10 years or more	203	52.4	13.0	
*Hours/week care giving (n* = *386)*				
<10 h	56	14.5	16.9	40.46***
10+ h	330	85.5	12.8	
*Help with personal care (n* = *387)*				
No	131	33.9	15.3	32.13***
Yes	256	66.1	12.5	
*Giving medicines? (n* = *387)*				
No	115	29.7	15.5	34.35***
Yes	272	70.3	12.6	
*Home design for caring (n* = *386)*				
Home design meets all, most of some needs	255	66.1	14.2	22.01***
Home design is totally inappropriate for caring	131	33.9	11.9	
*Effect of caring on health*—*no effect on health (n* = *387)*				
No	288	74.4	12.1	125.92***
Yes	99	25.6	17.4	
*Motivation for caring*—*no one else available (n* = *387)*				
No	188	48.6	14.4	16.58***
Yes	199	51.4	12.5	
*Motivation for caring*—*care recipient would not want anyone else to help (n* = *387)*				
No	185	47.8	14.4	15.64***
Yes	202	52.2	12.5	
*Effect of caring*—*time for leisure or social activity (n* = *387)*				
No	153	39.5	16.0	96.20***
Yes	234	60.5	11.7	
*Effect of caring*—*employment (n* = *387)*				
No	241	62.3	14.3	25.67***
Yes	146	37.7	11.9	
*Effect of caring*—*financial difficulties (n* = *386)*				
No	257	66.4	14.7	63.50***
Yes	129	33.3	10.9	
*Carer’s satisfaction with social care services (n* = *378)*				
Extremely, very or quite satisfied	225	59.5	14.5	34.77***
Neither satisfied nor dissatisfied, or dissatisfied	153	40.5	11.7	
*Completion of interview by telephone (n* = *387)*				
No, by face-to-face interview	336	86.8	13.53	1.30
Yes, by telephone	51	13.2	12.72	

Significance relates to the post hoc comparisons with Bonferroni correction to account for multiple comparisons

* *p* < 0.05; ** *p* < 0.01; *** *p* < 0.001

^a^One-way ANOVA

The responses by ASCOT-Carer domain are shown in Table 4. The majority of carers (93.2 %) reported quality of life at the ‘ideal state’ in one or more domain. The rating of each domain at the ideal state ranges from 20.7 % (*Space and time to be yourself, feeling encouraged and supported*) to 72.1 % (*Personal safety*). Almost half (49.1 %) of carers had some or high needs in the *Occupation* domain, whereas only 6.5 % reported that they felt less than adequately safe or not at all safe in the *Personal safety* domain.

**Table 4:** Responses to the ASCOT-Carer INT4 by domain

	Frequency	% (*n* = 387)
*Occupation*		
Ideal state	85	22.0
No needs	112	28.9
Some needs	158	40.8
High-level needs	32	8.3
Missing	0	0.0
*Control over daily life*		
Ideal state	101	26.1
No needs	143	36.9
Some needs	131	33.9
High-level needs	12	3.1
Missing	0	0.0
*Self*-*care*		
Ideal state	152	39.3
No needs	136	35.1
Some needs	67	17.3
High-level needs	32	8.3
Missing	0	0.0
*Personal safety*		
Ideal state	279	72.1
No needs	83	21.4
Some needs	17	4.4
High-level needs	8	2.1
Missing	0	0.0
*Social Participation*		
Ideal state	141	36.4
No needs	116	30.0
Some needs	98	25.3
High-level needs	31	8.0
Missing	1	0.3
*Space and time to be yourself*		
Ideal state	80	20.7
No needs	142	36.7
Some needs	136	35.1
High-level needs	29	7.5
Missing	0	0.0
*Feeling supported and encouraged*		
Ideal state	80	20.7
No needs	133	34.4
Some needs	111	28.7
High-level needs	61	15.8
Missing	2	0.4

**Fig. 1:**
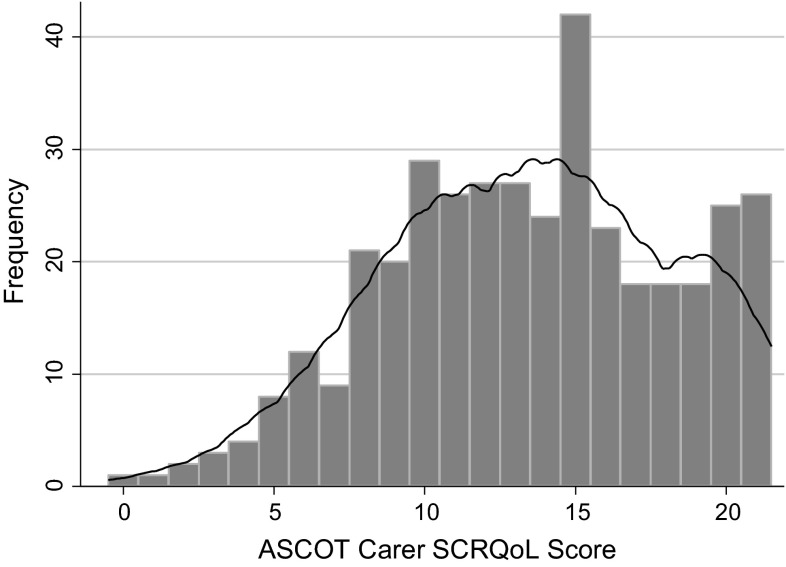
Distribution of the ASCOT-Carer social care-related quality of life scores (*n* = 384)

The overall ASCOT-Carer SCRQoL score has a negatively skewed and possibly bi-modal distribution (Fig. 1). The distribution indicates that there may be a ceiling effect at the upper end of the scale. The rate of missing values was low with less than 1 % (3) of respondents who had one or more missing values. This indicates that the questions are acceptable and feasible. Cronbach’s alpha for the ASCOT-Carer SCRQoL score was 0.87 (seven items). An alpha of 0.8–0.9 considered to be good [46], which indicates that the instrument has good internal consistency.

### Factor Structure

**Fig. 2:**
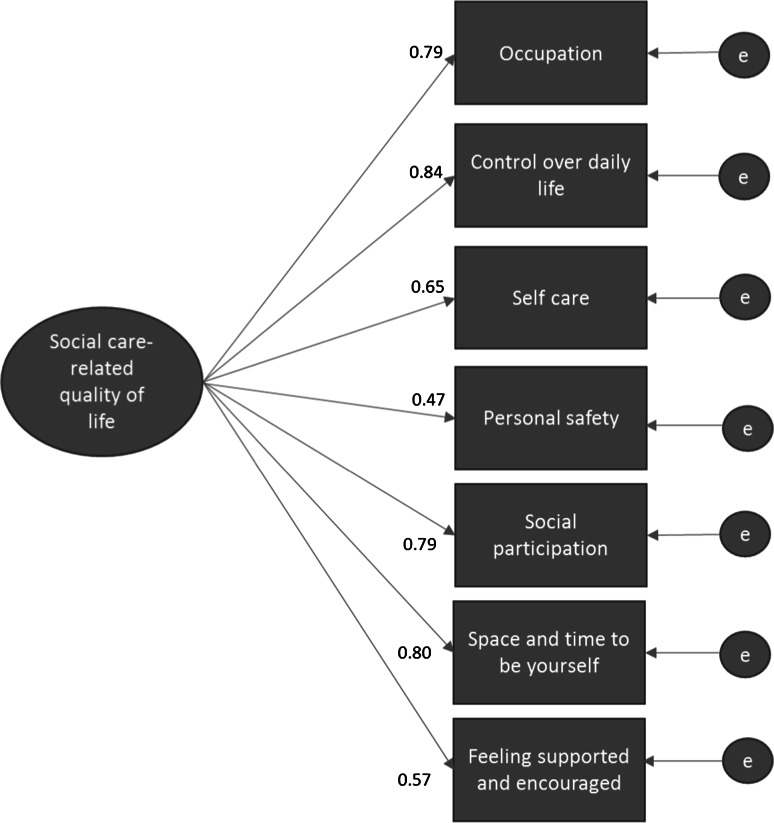
Standardised parameter estimates and squared multiple correlations for the one-factor structure of the seven ASCOT-Carer domains (*n* = 384) (Model 1)

**Fig. 3:**
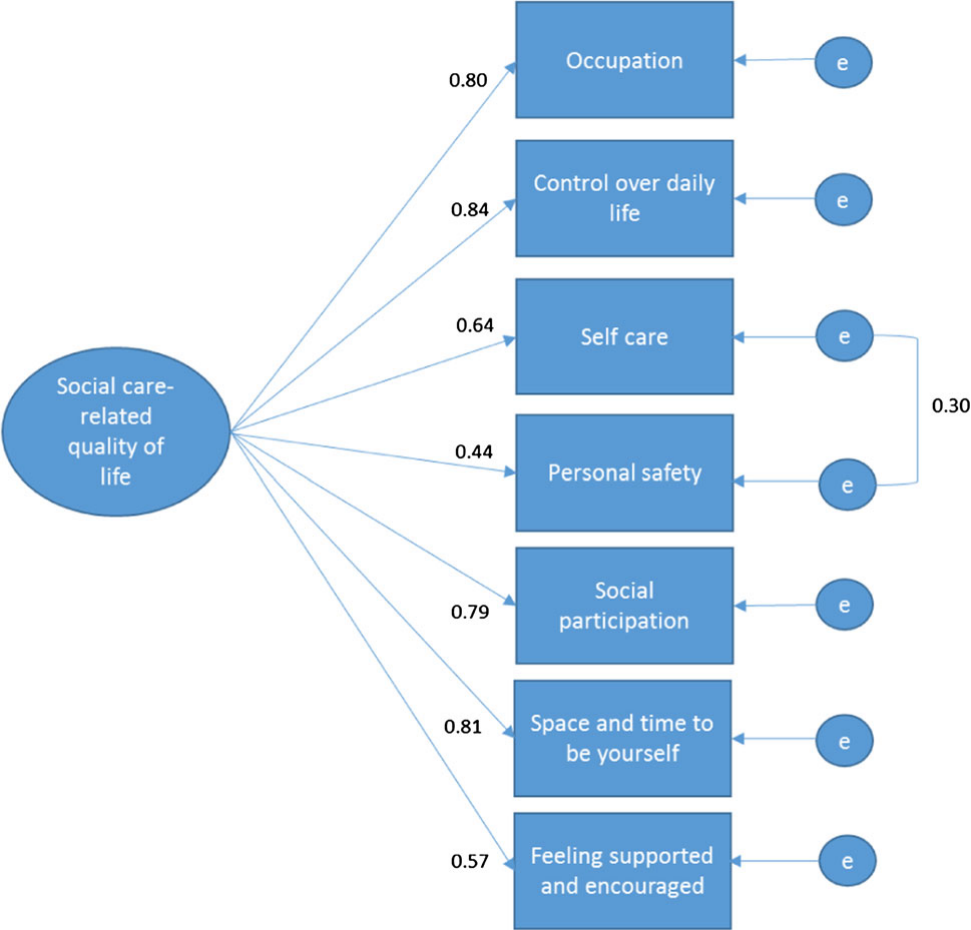
Standardised parameter estimates and squared multiple correlations for the one-factor structure of the seven ASCOT-Carer domains with correlated error term (*n* = 384) (Model 3)

The results of the confirmatory factor analysis are shown in Table 5, which shows that the overall goodness of fit Chi-square was significant for the hypothesised one-factor model (Model 1, Fig. 2). This suggests a lack of fit between the hypothesised model and the data. Other fit indices were also assessed due to the sensitivity of Chi-square in larger samples (≥200) [47]. These fit indices indicate adequate model fit following the standardised root mean squared residual (SRMR) and the CFI/TLI criteria of ≤0.08 and ≥0.95, respectively. However, the ≤0.06 criterion for the root mean square error of approximation (RMSEA) was not met and the modification indicated that freeing the covariance between the two error terms for *Self*-*care* and *Personal safety* would improve the model fit. Two alternative models to either omit the safety domain (Model 2) or free the path between *Self*-*care* and *Personal safety* (Model 3) were found to have better fit than the constrained model (see Table 5). Model 3 was preferred over Model 2 because of the face validity of the *Personal safety* domain and the significant improvement in model fit. All items loaded significantly at the 1 % level onto the single factor (ranging from 0.44 to 0.84, see Fig. 3). Change in Chi-square between the constrained (1) and non-constrained model (3) was significant (Δ*χ*^2^(1) = 33.6, *p* < 0.001).

**Table 5:** Confirmatory factor analysis of ASCOT-Carer INT4

	Model 1 (one factor)	Model 2 (one factor omits safety)	Model 3 (one factor with correlated error term)
*χ* ^2^	52.55	13.60	18.95
Degrees of freedom (*df*)	14	9	13
*p* value	<0.001	0.137	0.125
RMSEA (90 % CI)	0.085 (0.061–0.110)	0.036 (0.000–0.074)	0.035 (0.000–0.066)
SRMR	0.037	0.016	0.019
Comparative Fit Index (CFI)	0.969	0.996	0.995
Tucker–Lewis Index (TLI)	0.953	0.993	0.992
Coefficient of determination (CD)	0.901	0.899	0.900

### Construct Validity

Associations between ASCOT-Carer score and other related measures are shown in Table 6. As expected, the ASCOT-Carer score was significantly positively associated with EQ-5D and CES (preference weighted), as well as rating of quality of life on a single seven-item scale. There was a significant negative relationship between ASCOT-Carer and the CSI score. These relationships are congruent with the hypothesis that higher social care-related quality of life would be associated with more positive experiences of caregiving, better HRQoL and overall QoL, and lower reported carer strain.

**Table 6:** Bivariate correlation analysis of ASCOT-Carer INT4 and the EQ-5D, Carer Experience Scale (CES) preference-weighted, Carer Strain Index (CSI) and overall Quality of Life (QoL) (Pearson’s correlation coefficient)

	Mean (SD)	Correlation with ASCOT-Carer SCRQoL
ASCOT-Carer SCRQoL (*n* = 384)	13.4 (4.7)	–
EQ-5D (*n* = 382)	0.76 (0.3)	0.3430***
EQ-5D: mobility (*n* = 387)	1.3 (0.5)	−0.2138***
EQ-5D: self-care (*n* = 387)	1.1 (0.3)	−0.1260*
EQ-5D: usual activities (*n* = 387)	1.3 (0.5)	−0.1908***
EQ-5D: pain/discomfort (*n* = 386)	1.6 (0.6)	−0.2329***
EQ-5D: anxiety/depression (*n* = 384)	1.5 (0.6)	−0.3959***
Carer Experience Scale (CES) (*n* = 376)	68.7 (17.8)	0.5839***
Carer Strain Index (CSI) (*n* = 384)	6.4 (3.8)	−0.5933***
QoL (single item) (*n* = 384)	4.6 (1.0)	0.6169***

* *p* < 0.05; ** *p* < 0.01; *** *p* < 0.001

Univariate analysis of the characteristics hypothesised to be associated with ASCOT-Carer score (Table 2) are shown in Table 3. All hypothesised associations, with Bonferroni correction to account for multiple comparisons, reached significance except for carers’ age, duration of caregiving and survey administration mode (*p* ≥ 0.05). Multivariate regression analysis (Table 7)^2^ shows that, after controlling for other variables included in the model, 12 of the 25 hypothesised relationships reached significance at the 5 % level with one further relationship that indicated a trend towards significance (*p* < 0.1).

**Table 7:** OLS regression with ASCOT-Carer INT4 SCRQoL score as the outcome variable

Variable	Coefficient (B)	SE	Stand. Coefficient (β)	*p* value
Carer sex: male	0.61	0.34	0.06^	0.077
Carer aged 65+ years	−0.14	0.38	−0.02	
Carer in paid employment	0.69	0.42	0.07	
Carer’s health (rated as bad or very bad)^†^	−1.71	0.48	−0.14***	<0.001
UCLA three-item loneliness scale [38]	−0.61	0.1	−0.26***	<0.001
Cared-for person’s health (rated as bad or very bad)^†^	−1.03	0.39	−0.1**	0.009
Co-resident with cared-for person	−0.67	0.46	−0.06	
Cared-for person has short-term memory problem	0.12	0.39	0.01	
Cared-for person is disorientated	−0.65	0.43	−0.07	
Cared-for person has severely impaired cognitive skills	0.28	0.49	0.02	
Cared-for person has communication problems	−0.23	0.39	−0.02	
Frequent behaviour that the carer finds challenging	−1.38	0.61	−0.09*	0.024
Caregiving for ten or more years	−0.36	0.33	−0.04	
Hours of caring ≥10 h per week	−1.2	0.56	−0.09*	0.032
Helps cared-for person with personal care	−0.41	0.4	−0.04	
Helps cared-for person with medicines	−0.28	0.42	−0.03	
Home design does not meet all needs of carer	−0.21	0.36	−0.02	
No effect of caring on health	1.82	0.44	0.17***	<0.001
Reason for caring: no one else available	−0.24	0.34	−0.03	
Reason for caring: the care recipient would not want anyone else	−0.68	0.34	−0.07*	0.046
Caring has affected time for social and/or leisure activities	−1.51	0.39	−0.16***	<0.001
Caring has affected employment	−0.89	0.37	−0.09*	0.016
Caring has caused financial difficulties in the last 12 months	−0.86	0.38	−0.09*	0.025
Neither satisfied or dissatisfied, very or extremely dissatisfied with social care^††^	−1.48	0.34	−0.15***	<0.001
Interview completed by telephone^†††^	−0.99	0.49	−0.07*	0.042
Constant	21.48	0.79	–	–
*Model statistics*				
N	367			
AIC	1870.97			
*χ* ^2^	22.65***			
Adjusted *R* ^2^	0.596			

^ *p* < 0.1; * *p* < 0.05; ** *p* < 0.01, *** *p* < 0.001

^†^ Base category: rated as fair, good or very good

^††^ Base category: extremely, very or quite satisfied with social care services

^†††^ Base category: completed interview face-to-face

Positive relationships were observed for rating that caregiving had no effect on the carer’s health and for male (compared with female) carers. Negative associations were found for: poor health of carer; poor health of care recipient; higher self-reported social isolation and loneliness; carer and care recipient living together; frequent challenging behaviour by the care recipient; carer motivation for caregiving is that the care recipient would not want anyone else to look after him/her; more than 10 h of informal care undertaken per week; and completion of the interview by telephone compared with face-to-face. There are also negative associations with other reported negative impacts of caring, such as financial difficulties, caregiving affected employment and having less time for social or leisure activities. Finally, as would be expected since the ASCOT-Carer is designed to measure aspects of QoL targeted by social care support, a fair or poor rating of satisfaction with services was significantly related to lower QoL. The largest effects on the ASCOT-Carer score were observed for loneliness and isolation (*β* = −0.26), the effect of caring on social or leisure activities (*β* = −0.16), satisfaction with social care services (*β* = −0.15) and the effect of caring on health (*β* = −0.14), all of which relate either to aspects of caregiving that social care services may target (e.g. providing information and advice; support to enable carers to socialise or leave the home) or to the perceived quality and adequacy of services.

## Discussion

This study shows that the ASCOT-Carer is a unidimensional measure of the social care-related quality of life of unpaid carers of adults with physical disability, sensory impairment, mental health problems and intellectual disabilities in a valid and reliable way. The ASCOT-Carer has excellent feasibility with a very low percentage of non-response to the questions. The ASCOT-Carer INT4 has good internal consistency of responses, which indicates that it has high internal reliability. The factor analysis provides support for the findings of earlier work [20] by indicating that the seven items capture a single underlying factor of social care-related quality of life with covariance of error terms between *Self*-*care* and *Personal safety* domains. The path between these two domains may be justified by the conceptual link between the two constructs. Specifically, they both relate to sense of personal security, safety and care that may be at risk in particular types of caregiving situation: for example, high-intensity dementia caregiving. The covariance of error terms may, however, alternatively be due to a sequential ordering effect since *Personal safety* directly follows *Self*-*care* in the questionnaire, or associated with the marked ceiling effect in the *Personal safety* domain with 72 % of responses rated at the ideal state. Given the perceived need to retain the *Personal safety* domain for face validity, however, further work to explore these two domains would be justified.

The analysis presented in this article supports previous qualitative work on the domains of SCRQoL for carers [26, 29] to provide evidence of the construct validity of the ASCOT-Carer. The construct validity analysis demonstrates the expected relationships between ASCOT-Carer score and measures that capture related constructs. The weakest associations are observed between ASCOT-Carer score and the EQ-5D index and five individual EQ-5D dimensions. This would be expected since the EQ-5D captures the distinct (but related) construct of HRQoL, whereas SCRQoL deliberately omits overtly health-related domains to focus instead on other domains associated with the effect of social care on quality of life [21]. Moderate associations were observed for overall quality of life and the carer-specific measures of experience and burden. The ASCOT-Carer performs as expected, and the findings indicate that the measure captures a different construct to existing measures of carer strain, caring experience and health-related quality of life. Furthermore, the hypothesised relationships between SCRQoL and related measures or contextual factors reached significance in the univariate analysis in all except for two cases, and half of these relationships were also significant in multivariate analysis that controls for the other factors. In the multivariate analysis, the largest effects were observed for the perceived quality and adequacy of social care support, as well as factors (e.g. loneliness and isolation, impact of caring on health and social or leisure time) that social care services and policy aim to address. This indicates that the ASCOT-Carer measures what it is intended to measure, namely the aspects of quality of life related to concerns of carers that may be supported by social care service or policy interventions [26].

The ASCOT-Carer parallels the ASCOT for users of social care services, which is a preference-weighted measure of social care-related quality of life designed to be used in effectiveness and cost-effectiveness evaluations of social care policy and practice [21–23]. The ASCOT and ASCOT-Carer have three overlapping domains that are of concern to both social care service users with long-term conditions and their unpaid carers (i.e. *Occupation, Social participation* and *Control over daily life*). Although the ASCOT and ASCOT-Carer have been developed based on the distinct concerns of users and carers, they both measure social care-related quality of life and may therefore be used in conjunction with providing an estimate of SCRQoL for an individual and their carer(s). Further work to establish preference weights for the ASCOT-Carer and to map how the two preference-weighted measures complement each other may support the combined use of these two measures in evaluation of the wider impact of policy and practice on both people with long-term conditions and their carers.

The strength of this study is the wide range of variables included in the data set to capture characteristics of the carer, the care recipient and the caregiving situation, which has enabled a comprehensive evaluation of construct validity. Although three hypothesised relationships were not observed, in general the findings support the use of the ASCOT-Carer to measure social care-related quality of life in a diverse group of carers. Nevertheless, the study has some limitations to be highlighted. First, the study presented here does not directly assess the responsiveness of the measure to changes in social care services. This could be addressed by testing for a positive relationship between ASCOT-Carer score and intensity of service use, while controlling for amount of caregiving as a proxy for social care need. Due to incomplete data provided by local authorities, the data set analysed in this article does not include a robust measure of the intensity of social care service support for the carer and/or care recipient. Further work to establish the responsiveness of the ASCOT-Carer to social care interventions, as well as the sensitivity of the instrument to change over time, would, therefore, be valuable. Second, the small number of respondents restricted analyses to the whole sample rather than subgroup analyses that may have been of interest. For example, carers of people with dementia or an analysis of older (≥65 years) compared with younger carers may be instructive given their different needs. Third, the findings indicate that there may be a weak bias towards lower reporting of ASCOT-Carer SCRQoL when data collection is by telephone rather than face-to-face interview. This effect should be considered in future work that draws on a mix of survey administration modes. Finally, this study has only explored reliability in terms of the internal consistency of the measure. Further work to establish the test–retest reliability of the measure is warranted.

## Conclusion

This study has provided evidence for the unidimensional factor structure of the ASCOT-Carer INT4 scale and internal consistency of responses. It has also provided good evidence for the construct validity of the measure for a diverse group of carers. These findings are encouraging and support the use of ASCOT-Carer INT4 to measure the outcomes for carers of social care interventions and policy. Further work is required to explore the relationship between the *Personal safety* and *Self*-*care* domains and to explore the properties of the measure for subgroups of carers, for example carers of people with dementia.

## Footnotes

1 An exploratory factor analysis conducted on the sample presented in this article also proposed a one-factor (single scale) solution for the seven-item, four response-level version. An appendix outlining this analysis is available upon request to the corresponding author.

2 The residuals are normally distributed (Shapiro–Wilk W test for normality (*W* = 0.996 *V* = 0.971 *Z* = −0.070 *p* = 0.528). The variance of the residuals is homogenous (White’s test for heteroskedasticity (*χ*²(326) = 339.8, *p* = 0.288). There is no evidence for omitted variable bias (Ramsey-reset test for omitted variables (*F*(3,338) = 0.76, *p* = 0.517).
